# Indiana Hospital for the Insane

**Published:** 1852-01

**Authors:** 


### Part 4.—Editorial.

### ARTICLE I.

### INDIANA HOSPITAL FOR THE INSANE.

In a preceding number of the Journal we noticed the reception of the Report of this Institution for 1850, with a copy of the report of a committee appointed by the State Legislature to investigate charges made against some of its officers, which we promised to notice and to make some historical remarks in reference to the establishment; which up to this time have been neglected. The reception of the report of the institution for 1851 again reminds us of that promise and though limited for space we will endeavor to redeem it. If it becomes necessary in giving this history for us frequently to refer to the part we took in originating, planning and building up the institution we hope it will not be regarded as immodest, as no record has yet been published that furnished an accurate account of its inception and early management.

The originie of the Indiana Hospital for the Insane was in the darkest hour of Indiana's pecuniary history. It was when her own treasury notes, bearing six per cent. per annum interest and receivable for State revenue were only worth fifty cents on the dollar. It was when the State debt like a huge mountain whose top was out of sight rested as an incubus upon her, and paralyzed to irresolution the energies of her stoutest hearts. As of necessity under such circumstances, its progress was slow. In December, 1840, a memorial from Dr. Isaac Fisher and myself, then of Attica, was sent to the Legislature, upon which Dr. James Ritchey, now one of the commissioners of the institution, and from this time forward one of its most efficient and devoted friends, made a report, from a standing committee of the Senate, in which he ably set forth the necessity of the measure. The next autumn I published an article in the Wabash Express urging upon the people its necessity, and the winter following sent up another memorial to the Legislature which was, with one from Dr. Matthews, referred, and again favorably reported upon by Dr. Ritchey, who introduced a resolution instructing the Governor to correspond to obtain information upon the subject. In pursuance of this, Governor Bigger employed an architect, Mr. I. P. Smith, of New Albany, Ia., to visit the Ohio Lunatic Asylum, and draw a plan for such an institution as he thought Indiana required, which plan was laid before the Legislature the following winter, but received no further attention.

I again laid a memorial before the Legislature, urging the necessity of immediate action upon the subject, which was referred to the committee on Education, which committee I addressed before a public audience, by special invitation, on the 25th of December, 1843. In this address, which was published at the time, and by request of the committee having charge of the subject re-published the winter following, I set forth the necessity of such an institution and the obligation of the State to provide one, and urged that a tax of one cent on each one hundred dollars valuation of property be levied then, to raise a fund with which to erect buildings.

The committee of the Senate consented to reconsider a report that had been drawn up against the measure, and by the efforts of Drs. Ritchey and Cornett, reported in favor of the tax being levied, which report was adopted by the Senate, and the revenue bill was so passed as to impose the tax. This tax was afterwards continued from year to year.

The following winter, 1844–5, three commissioners were appointed to purchase a site and present a plan for a building.

Dr. L. Dunlap, James Blake Esq., and myself constituting the board.

After a visit to similar institutions in the Eastern States, I reported to this board the results of my observations, with the description of a plan for a building, which report was published in the first report of the commissioners to the Legislature of 1845--6. That Legislature adopted the plan of building I had recommended, and which the board had employed Mr. John Elder, architect, to make drawings of, and, also, authorized its immediate erection. Materials were purchased and the basement story erected the following summer and fall under my care, having been appointed Superintendant. During the summer of 1849, the walls were erected, and it was then contemplated that a part of the building might be furnished early the following year so that it might go into operation. I gave the board notice that I should resign the following year as I did not design to continue superintendent after the institution was ready to receive patients. A law was passed the winter following for the government of the institution and providing for the mode of admitting patients. In July, 1848, R. J. Patterson, M. D., the present superintendent, entered upon his duties.

Since this time the institution has annually been sending forth the triumphs of its power to heal, in its numerous patients that have been returned to their families and friends clothed and in their right minds.

Although there may have been some errors in the management of the institution, (it is human to err,) which could be seized upon and used as weapons in the hands of opponents to its officers to injure them, as would appear from the investigation that was had, the good it has done, its sacred character, its benevolent object, its mission of mercy, should guard and protect it against any exaggeration of those errors, any unfounded accusation, or any malicious opposition.

The investigation of the charges appears to have been thorough, and from the honorable character of the gentlemen composing the committee, we infer that it is worthy of confidence.

The report of the committee finds no blame to attach to the commissioners, excuses all the errors of the superintendent that came to their knowledge, on the ground of inexperienced attendants, and says: "The manner in which the duties of the Assistant Physician, Matron, and Steward are discharged is worthy of all praise and commendation."

We are exceedingly sorry to see the law prohibiting the officers of the institution from trafficking with it disregarded, as the law is the only security that the State has, against the subordinate officers of the institution using their patronage in trade to secure the favor of those who enjoy the appointing and removing power, which, if so used, will as certainly lead to abuse. No steward holding under a board that have the power of removing him, if members of that board are engaged in trade with the institution, will be likely to be perfectly independant in the discharge of his duties. And if the steward was allowed to speculate in furnishing the establishment his perquisites would be easily swelled to thousands. We hope the people will insist upon the observance of this wholesome and salutary law, and extend its provisions to the other Benevolent Institutions.

The reports of the institution show a fair proportion of cures and we have no doubt but that it is entirely worthy of the confidence of those having charge of insane persons.

We have observed the manner in which the design of the building has been carried out, and are gratified to see that there has been but little alteration from the original plan. While it is to be expected that in minor arrangements about the conveniences of different parts of so extensive an establishment there would be differences of opinion, still the propriety of the arrangements generally have been sanctioned, even with commendation.

We can but regret, however, the short-sighted policy which has led to the abandonment of the original plan of heating and ventilating the establishment; a plan much more expensive and incomparably inferior in point of health and comfort, and even decency, as we shall proceed to demonstrate, having been substituted.

We set it down as established:

1st. That the object and great advantage of heating by hot air, is the constant change of air kept up in the apartments, by which they are kept sweet and wholesome.

2nd. That without a continual and rapid circulation of air from the heating apparatus to the apartments heated, a large amount of heat must be wasted; increasing very much the expense of heating, which, in an establishment of the size of this, is a very important item.

And we unhesitatingly assert, that without a *forced* or *active* ventilation, such as was originally designed, neither of these objects can be properly attained.

That our readers may understand, we will give the original plan in brief.

To heat the patients' wards a steam boiler was to be placed in a back building for each wing, connected with which a sufficient series of pipes were to be placed in a hot-air chamber so as to be kept full of steam, and consequently at a temperature of about 212° Farenheit, to give off heat enough to warm the whole wing. The air thus heated coming in freely at the lower part of the chamber from out doors and passing up to each ward through a flue 2 by 4 feet in the clear.

After very expensive experiments in varying the plan for heating thus described, those in charge have returned to it, with the exception that they make additional hot-air chambers.

But the heating apparatus thus described is very imperfect withuot the system of ventilation which was an essential appendage to it in the original plan, but which has unfortunately been abandoned. The abandonment was said to be in consequence of a failure to work upon first trial, which failure depended upon a slight mistake that would have been easily remedied, as we will presently show.

The plan of ventilation was to have a flue from each room, (which flues are already in the walls,) to extend to the fire that heats the boiler, so as to have a direct draft from every patient's room in the building. The mistake referred to, was in the termination of the collection of flues from the rooms, which could easily have been made under the fire, instead of being in a large chimney or shaft, by which a strong draught would be produced. This would exhaust the air from each room in the wards, as has been abundantly tested by the McLean Asylum near Boston, and at the Blockly Almshouse, Philadelphia.

Now, in as much as the greatest difficulty of heating, by hot air, depends upon the presence of the air already in the room preventing the hot air from entering it, this forced ventilation, by which the cold air is drawn off to give room for, and even call in the hot air, is an essential point. Without it, a much greater amount of fire will be required to make the apartments comfortably warm, and hence the increased expense on account of its abandonment.

By the plan of ventilation originally designed, and for which the flues were all prepared when I le ftthe Institution, the cold air was to enter the hot-air chamber where enough steam pipes could be placed to heat it as rapidly as desired; when heated it would ascend through the large flues to the hall in each ward, from which it would be called into the different rooms and apartments through open transums over the doors, by the exhausting force of the ventillating flues, which were to be regulated by registers at their openings; and as fast as the air become cool it would take the lower stratum in the room and be drawn off by the flue near the floor, to give place for a fresh supply of hot air. By adjusting these registers, the different rooms could be cooled or heated at pleasure, by more or less rapidly drawing off the cold air and calling in the heated. This would have rendered every part of the establishment pleasant, and free from all offensive odors.

How men, who at one time seemed to appreciate the plan, could abandon it at once, and spend large amounts uselessly experimenting upon other and untried projects, without sending to examine or even enquiring in reference to a similar plan then in successful operation at Boston, is a mystery that we are unable to solve.

But the commissioners and superintendent together did even so, the result of which is that a passive ventilation in cold flues is all that is had; the results of which again are a difficult and imperfect distribution of the hot air to the apartments; a difficulty in getting a draught from the hot-air chamber; an imperfect change of air by which the apartments are filled to a considerable extent, with the effluvia from patients, (always vile,) and an increased expense for fuel.

But this is not all. One ward, where there is the most filth, is actually heated by the passage through its entire length of a large steam pipe! thus heating over and over again the foul air contained in it, without any except an accidental change of air. This mode of heating without ventilating in the ward for the worst class of patients renders it so foul a place that it is with difficulty that suitable persons can be induced to live in it as attendants. We hope a reform, at least in this ward, will soon be had.

The want of a system or plan in the changes that have been made under the present Superintendent, has led to a constant series of building and pulling down again at great expense.

As it is recommended to extend the buildings immediately, we would advise the employment of Dr. Bell, of Boston, or some other such person, to make a design for the additions, and enjoin upon the architect to follow out his plans, in all their details, he having system, experience, extensive and correct information upon the subject, with a sound and discriminating judgment. But as the Superintendent, in his report for 1848, says he finds the building amply large for the accommodation of two hundred patients, and only 145 having been in, at any one time yet, we should think it would be well to defer the proposed addition until the present building was full.

We had designed noticing several points in the reports, but are compelled to defer it to the next number for want of room. The large number of our subscribers in all parts of Indiana, who are directly interested in this Institution, and the importance of the subject of heating and ventilating, must be our apology for spending so much time in referring to it.

---

